# Coexistence of a Calcium-Sensing Receptor Mutation and Primary Hyperparathyroidism

**DOI:** 10.7759/cureus.46980

**Published:** 2023-10-13

**Authors:** Peyton Russell, Mc Anto Antony

**Affiliations:** 1 Endocrinology and Metabolism, Medical University of South Carolina, Charleston, USA; 2 Endocrinology, AnMed Health, Anderson, USA

**Keywords:** calcium-sensing receptor gene, casr mutation, parathyroid gland adenoma, familial hypocalciuric hypercalcemia, hypercalcemia, primary hyperparathyroidism

## Abstract

Primary hyperparathyroidism (PHPT) and familial hypocalciuric hypercalcemia (FHH) are the main differential diagnoses in a patient presenting with parathyroid hormone (PTH)-mediated hypercalcemia. PHPT is most often caused by a single-gland parathyroid adenoma and FHH is the result of an inactivating mutation of the calcium-sensing receptor (CaSR) gene.

In this paper, we present a unique case of the co-existence of an inactivating CaSR gene mutation and PHPT due to a single-gland parathyroid adenoma. The patient is a 67-year-old female with a history of recurrent nephrolithiasis who presented with hypercalcemia, elevated PTH level, and hypocalciuria. As a result of the patient’s hypocalciuria, familial hypocalciuric hypercalcemia was suspected, and genetic testing was pursued. CaSR gene analysis revealed a heterogeneous inactivating mutation of the CaSR gene. Additionally, nuclear imaging with technetium sestamibi revealed a large focus of activity on the right side of the neck suspicious of a parathyroid adenoma. This was resected and confirmed to be a hypercellular parathyroid adenoma. Two years after her surgery, the patient continues to have normal calcium levels with no further episodes of nephrolithiasis. She is currently undergoing treatment for osteoporosis and is being periodically monitored for recurrence of hypercalcemia due to the presence of the inactivating CaSR gene mutation. This case highlights an exceedingly rare case of a patient with both an inactivating CaSR gene mutation and PHPT due to a single parathyroid adenoma, and it underscores the importance of further research to determine any potential relationship between the two.

## Introduction

Primary hyperparathyroidism (PHPT) is the most common cause of hypercalcemia and is usually caused by a single-gland parathyroid adenoma. Asymptomatic hypercalcemia is the most common manifestation of PHPT, but the disease can be complicated by bone demineralization and kidney injury in the setting of nephrolithiasis and nephrocalcinosis [1].

PHPT is one of the most common endocrine disorders with an estimated prevalence of one to seven cases per 1,000 adults [1]. Determining incidence has been difficult due to global heterogeneity in screening methods, case definitions, the populations studied, and annual fluctuations. However, it is understood that it is a disease process that occurs significantly more frequently in women than men, with an average incidence of 66 per 100,000 women. This is in contrast to approximately 25 cases per 100,000 men [1]. Age is an additional factor that is related to an increased prevalence of PHPT. In both men and women less than age 50, prevalence has been found to be 36 and 80 per 100,000 individuals, respectively. In both men and women between ages 50 and 59, prevalence was found to be 36 and 80 per 100,000 individuals. In men and women aged from 70 to 79, prevalence was found to be 95 and 196 per 100,000 individuals, respectively [1].

An elevated corrected (for serum albumin) serum calcium level that is incidentally detected on routine laboratory testing is usually the first indication of the presence of a hypercalcemic disorder and if the corresponding serum intact parathyroid hormone level (iPTH) is inappropriately normal or elevated, it points toward a parathyroid hormone (PTH)-mediated etiology of the hypercalcemia. In most cases of PHPT, the serum PTH levels are >65 pg/ml (normal range: 15-65 pg/ml). However, there is another entity known as normocalcemia PHPT in which the serum calcium level is within the normal range and PTH is elevated [2]. In addition to the evaluation of serum calcium, serum phosphorous and vitamin D can help confirm the diagnosis [3]. With elevated PTH levels, hypercalcemia can ensue via the PTH-mediated action at the level of the bone where it causes increased bone resorption. At the level of the distal tubule of kidneys, PTH not only causes increased calcium reabsorption but also enhances the increased production of 1,25-dihydrocholecalciferol, which subsequently acts at the level of the intestine to increase calcium absorption. PTH also decreases the expression of sodium-phosphate co-transporters in the kidney leading to urinary phosphate loss and hypophosphatemia [4]. In some severe cases, patients with PHPT will also have low or deficient levels of vitamin D [5]. This is thought to be the result of increased PTH-mediated 1-alpha hydroxylase activity in the kidneys, thus increasing the conversion of 25-hydroxyvitamin D to 1,25-dihydroxyvitamin D [1,25(OH)2D]. Elevated levels of 1,25(OH)2D are thought to suppress the production of activated vitamin D in the skin and liver [6]. Complications that may arise from prolonged elevations of PTH include osteoporosis, vertebral fractures, and nephrolithiasis [3].

In 80-90% of cases, PHPT is the result of a single parathyroid adenoma. Four-gland hyperplasia causes approximately 10-15%, multiple adenomas cause 5%, and parathyroid malignancies cause <1% of cases [7]. They can also be present in familial endocrinopathies such as multiple endocrine neoplasia (MEN) 1, 2A, and isolated familial hyperparathyroidism [8]. In rare instances, PHPT has been reported in the setting of an inactivating mutation in the calcium-sensing receptor (CaSR) gene, resulting in PTH-mediated hypercalcemia [9]. The CaSR gene is a G-coupled-protein receptor normally expressed in the parathyroids and kidneys and allows for the regulation of PTH secretion and renal calcium reabsorption based on the detection of extracellular calcium. Inactivating mutations of this gene prevent the body from sensing normal calcium levels, resulting in the release of increased PTH and increased calcium reabsorption in the kidneys [10]. There is currently limited data on the prevalence of CaSR mutations and no current reports on the population incidence of CaSR mutations in the setting of PHPT. However, there are other reports of inactivating CaSR mutations occurring in the setting of familial benign hypercalcemia, neonatal severe hyperparathyroidism, familial hypocalciuric hypercalcemia, and Bartter syndrome [10]. Identification of a CaSR mutation is based on genetic testing, with germline heterozygous and homozygous loss-of-function CaSR mutations having been reported in patients with adult-onset PHPT [8].

A significant relationship has not been found between inactivating CaSR mutations and parathyroid tumors in sporadic PHPT. However, it has been documented that CaSR expression is reduced in most hyperplastic and adenomatous tumors [8]. In this paper, we present a unique case of the co-existence of an inactivating CaSR mutation and PHPT in the setting of a single adenoma.

## Case presentation

A 67-year-old Caucasian female with a past medical history of post-surgical hypothyroidism, osteoporosis, and hypertension was referred to our endocrinology clinic in March 2020 for further evaluation of hypercalcemia and elevated PTH levels. As per records, the first documented hypercalcemia was 10.1 mg/dl (8.6-10.0 mg/dl) in November 2019. Her first documented PTH was 264.1 pg/ml (15-65 pg/ml), which was measured in February of 2020. Repeat lab testing revealed high corrected serum calcium 10.5 mg/dl, high PTH 119.4 pg/ml, and normal vitamin D3 33.9 ng/ml (>30 ng/ml). Clinically, the patient was asymptomatic but reported a history of recurrent nephrolithiasis. No stone analyses were ever completed. Twenty-four-hour urine calcium was low at 88 mg/24 hour (100-300 mg/24hr) with corresponding 24-hour urine creatinine of 740 mg/24 hr (740-1570 mg/24 hr) and urine volume of 1175 cc. Calcium to creatinine clearance ratio was 0.01. A dual X-ray absorptiometry (DEXA) scan performed at our radiology center showed progressive worsening from borderline osteopenia in March 2016 to osteopenia at multiple sites in May 2018, to osteoporosis in the left femoral neck and left distal radius in August 2020. Due to worsening DEXA scan findings and a history of recurrent nephrolithiasis, the patient was started on Prolia injections and underwent further evaluation, including a genetic analysis for CaSR mutations.

CaSR gene analysis revealed a heterogeneous mutation of uncertain significance at nucleotide position c.2777 in Exon 7 of the CaSR gene. This nucleotide change is predicted to result in an amino acid change from glutamine to arginine at position 926 in the CaSR protein (p. Q926R). The above mutation is reported to be present at a very low frequency in the ExAC (Exome Aggregation Consortium) and gnomAD (Genome Aggregation Database) database. The patient instructed her children to undergo genetic testing for CaSR mutations. Her daughter was found to have no mutations but her son could not be reached for follow-up.

**Figure 1:**
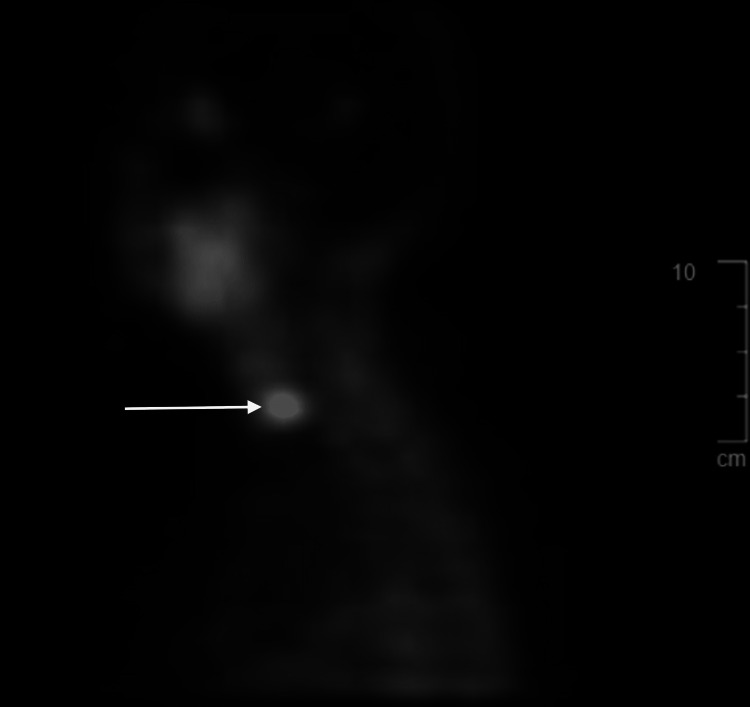
Sagittal plane of sestamibi scan indicating single parathyroid adenoma

**Figure 2:**
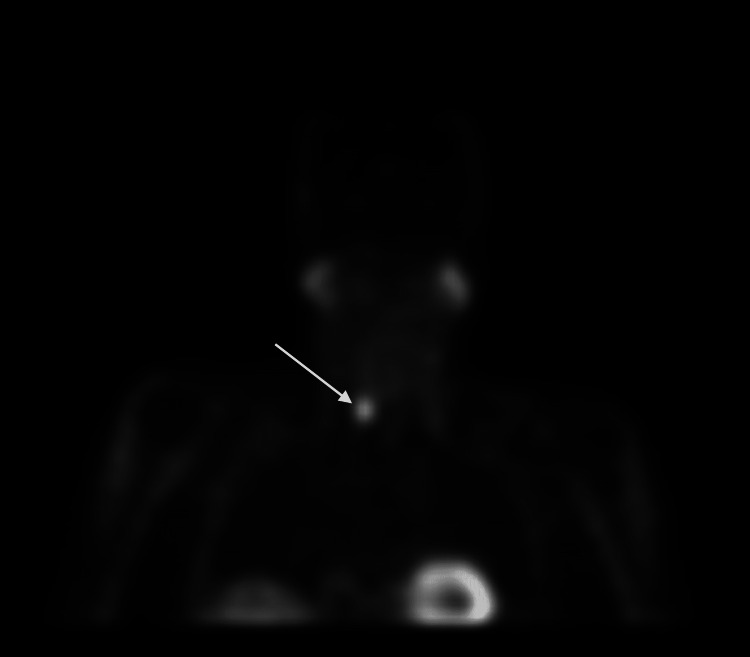
Coronal plane of sestamibi scan indicating single parathyroid adenoma

As part of the patient's continued workup, a parathyroid ultrasound revealed a 1.3 x 0.5 x 1.1 cm hypoechoic lesion present outside the right thyroid lobe. A nuclear medicine single-photon emission computed tomography (SPECT) scan using technetium sestamibi revealed a large focus of activity on the right side of the neck suspicious for a parathyroid adenoma (Figures 1, 2). The patient underwent bilateral cervical neck exploration in October 2020 and was found to have an enlarged right superior parathyroid adenoma that was resected. The intra-op PTH level fell from 175 to 41 pg/ml. Final pathology revealed a 1.5 cm hypercellular parathyroid gland weighing 0.58 grams. In December 2020, repeat calcium levels were found to be corrected at 9.6 mg/dl and PTH was normal at 62.3 pg/ml. It has been two years since the parathyroid surgery and the corrected serum calcium levels have remained consistently within the normal range, although increases in her calcium supplements have been required. She continued her Prolia injections until October 2022 and then switched to oral alendronate secondary to side effects. The most recent DEXA scan in August 2022 showed an improvement from osteoporosis to osteopenia at the level of the left femoral neck stable osteopenia in the lumbar spine and stable osteoporosis in the left wrist (Table 1). To date, the patient has not suffered any osteoporotic fractures and her nephrolithiasis resolved after her parathyroidectomy.

**Table 1:** DEXA scan and medication data DEXA: dual X-ray absorptiometry

Year	Medications Initiated/Held	Lumbar Spine Bone Mineral Density (BMP)	Lumbar Spine T-Score	Left Femoral Neck BMD	Left Femoral Neck T-Score	Right Femoral Neck BMD	Right Femoral Neck T-Score	Left Radius BMD	Left Radius T-Score
2018	Alendronate 70 mg/weekly	1.057 g/cm^2^	-1.1	0.737 g/cm^2^	-2.2	0.716 g/cm^2^	-2.3	0.463 g/cm^2^	-3.5
2020	Ergocalciferol 50000 IU/week Alendronate held	0.947 g/cm^2^	-1.8	0.684 g/cm^2^	-2.5	0.708 g/cm^2^	-2.4	0.641 g/cm^2^	-2.7
2021	Prolia initiated								
2022	Prolia stopped due to side effects; Alendronate 70 mg/weekly reinitiated, calcium 650 mg/vitamin D 12.5 mcg initiated	1.014 g/cm^2^	-1.5	0.744 g/cm^2^	-2.1	0.718 g/cm^2^	-2.3	0.648 g/cm^2^	-2.6

## Discussion

PHPT is most commonly caused by a single adenoma such as in the case described above. Management is generally curative with the removal of the affected parathyroid gland or glands. The rare presence of the CaSR inactivating mutation has so far not affected the post-surgical outcome of our patient, and she has remained largely asymptomatic with normocalcemia. However, it will be pertinent to monitor the patient’s calcium and intact PTH levels periodically to determine the clinical significance of the CaSR gene mutations. Although the patient’s DEXA scan showed a progressive worsening from osteopenia to osteoporosis, there has been a good improvement with a reversal back to the osteopenic range noted on her most recent DEXA scan as mentioned above. Besides monitoring for hypercalcemia recurrence, her bone density will also undergo timely monitoring to assess the treatment response.

Up to 10% of patients with PHPT can have a germline mutation that can impact calcium levels. The clinical indication for genetic testing for patients with PHTP is usually the result of PHTP occurring before age 45, multiglandular disease, and carcinomas or atypical hyperplasia of the parathyroid [11]. Our patient’s mutation at Q926R has been previously reported in patients with familial hypocalciuric hypercalcemia (FHH) and has been shown to cause decreased maximal response to calcium ions [9]. Although it is possible that our case is one of PHPT and FHH in the setting of a CaSR mutation, FHH is not associated with adenoma formation, and it would be unexpected for our patient’s hypercalcemia to respond to parathyroidectomy if she had FHH [10]. Although family history is usually positive in patients with FHH, our patient had only one other family member tested. As a result, a complete family history is unknown and FHH cannot be completely ruled out. One notable study found that in four patients with FHH/PHPT diagnostic overlap due to CaSR mutations and adenomas, all patients responded to surgery, just as ours did [12].

Currently, there is limited evidence that the CaSR gene plays a role in the formation of parathyroid adenomas, and any potential mechanism is not well understood [10]. A recent study found epigenetic changes and hypermethylation of the CaSR gene in cells of sporadic adenomas [13]. While the said study did not evaluate patients with CaSR gene mutations, it highlights a potential relationship between the CaSR gene alterations and adenoma formation. Furthermore, one article hypothesized that decreased functioning CaSR receptors would result in the proliferation of parathyroid cells [14]. This is an area of ongoing research and our case further displays the possibility of a relationship between CaSR mutations and parathyroid adenomas.

Our patient had one child tested for CaSR mutations, and that child was negative. Testing of siblings, other children, or any family members with hypercalcemia would be interesting to indicate whether our patient’s mutation variant is related to disease status and a possible genetic disease such as FHH. Further familial genetic testing would also allow for the formation and analysis of a kindred. It should be noted that it is possible the mutation found is a sporadic or non-familial germline mutation, which has been found to impact CaSR, MEN1, and CDC73 genes [11]. With the current genetic analysis of only one child, it cannot be determined if this case is familial or sporadic.

## Conclusions

The occurrence of PHTH due to a single adenoma in addition to the presence of an inactivating CaSR mutation is an exceedingly rare event. The clinical description presented here adds such a case to the literature. This case additionally highlights the need to understand any potential relationship between CaSR mutations and parathyroid tumors.
